# The Importance of the *Physcomitrium patens* Genome in the Evolutionary Genomics of Terrestrial Plants

**DOI:** 10.3390/plants15081261

**Published:** 2026-04-20

**Authors:** Anderson Franco da Cruz Lima, Wellington Bruno dos Santos Alves, Letícia Fernanda Presotti Matos, Yasmin Jansen Araujo, Michele Gomes de Morais, Giovanna Melo Nishitani, Stephan Machado Dohms, Marcelo Henrique Soller Ramada

**Affiliations:** 1 Programa de Pós-Graduação em Ciências Genômicas e Biotecnologia, Universidade Católica de Brasília, Brasília 71966-700, Brazil; anderson.lima@a.ucb.br (A.F.d.C.L.); leticia.fernanda@a.ucb.br (L.F.P.M.); yasminjaraujo@gmail.com (Y.J.A.); michele.morais@a.ucb.br (M.G.d.M.); giovanna.nishitani@a.ucb.br (G.M.N.); stephan.dohms@p.ucb.br (S.M.D.); 2 Programa de Pesquisa e Extensão (PROERT), Universidade Católica de Brasília, Brasília 71966-700, Brazil; wellington.bruno@a.ucb.br; 3 Programa de Pós-Graduação em Gerontologia, Universidade Católica de Brasília, Brasília 71966-700, Brazil

**Keywords:** moss, bryophyte genomics, model organisms, plant evolution, gene expression, plant biotechnology, *Physcomitrium patens*

## Abstract

Mosses (Bryophyta) comprises a group of terrestrial plants that colonized land more than 450 million years ago that play fundamental ecological and evolutionary roles, particularly in polar and peatland ecosystems. The sequencing of *Physcomitrium patens* marked a milestone in bryophyte genomics, establishing mosses as model organisms for evolutionary and functional studies. However, the recent advent of next-generation sequencing technologies has broadened genomic exploration beyond *P. patens*, unveiling the genetic diversity of additional bryophyte species. Notably, the genomes of *Sphagnum fallax*, *Sphagnum magellanicum*, the liverwort *Marchantia polymorpha* and hornworts from *Athoceros* genus have provided new insights into carbon fixation mechanisms, ecological adaptations, and lineage-specific evolutionary traits. These advances have enabled large-scale comparative analyses and expanded the understanding of conserved and divergent genomic features among bryophytes. The integration of these datasets into public databases such as Phytozome and NCBI Genome has created a robust framework for investigating plant genome evolution and biotechnological potential. Altogether, the expanding genomic landscape of bryophytes reveals their remarkable evolutionary plasticity and underscores their importance as key models for studying adaptation, metabolism, and genomic innovation in terrestrial plants.

## 1. Introduction

Mosses (Bryophyta) comprise the most abundant representative of the bryophytes and are non-vascular terrestrial plants that play fundamental ecological roles, particularly in sensitive and cold ecosystems such as boreal, alpine, and Antarctic ecosystems [1,2,3]. Their ability to colonize inhospitable surfaces, retain water and nutrients, and participate in carbon cycling and soil formation confers upon them an ecological importance [1,4,5]. The global distribution of mosses reflects their high adaptive plasticity and resilience to extreme environmental conditions. These traits make them ideal models for studying molecular mechanisms associated with tolerance to abiotic stress, including desiccation, low temperature, high radiation, and osmotic variation, and this is one of the reasons that make mosses model organisms [6,7].

Along with other lineages of the bryophyte group, studies on moss genomes have revealed key elements involved in the evolutionary transition of plants from aquatic to terrestrial environments [8,9]. Moreover, mosses exhibit a haplodiplontic life cycle with a dominant gametophytic phase, which facilitates functional genetic analyses. In particular, *Physcomitrella patens* (Hedw.) Bruch & Schimp., recently reclassified as *Physcomitrium patens* (Hedw.) Mitt., has emerged as a biological model for plant genomics and biotechnology due to its high efficiency in homologous recombination, a rare feature among plants [10].

The genome sequencing of *P. patens* was a milestone in evolutionary botany, enabling comparisons with angiosperms and revealing that approximately two-thirds of its genes are orthologous to those of vascular plants. This genome unveiled not only genes involved in stress responses but also gene duplications and genomic structures that are not observed in other bryophyte groups, consolidating the species as a window into the evolutionary past of terrestrial plants [11].

Therefore, this work addresses a critical gap by delivering a comprehensive synthesis of the current landscape of moss genomics, from the foundational sequencing of *P. patens* to the rapidly expanding genomic resources now available, and by outlining their biotechnological potential and phylogenomic relationships to major lineages of land plants [12].

## 2. *Physcomitrium patens* as a Model Organism

### 2.1. Model Moss and Its Applications

For a century, the moss *P. patens* has been used as an experimental model organism in plant biology, particularly for its unique combination of genetic, physiological, and experimental traits that distinguish it from other land plants [10]. This organism belongs to the Funariaceae family (Order Funariales) and thrives mostly on wet soils in damp areas of temperate climate zones in Europe, North America, and East Asia. Along with other moss species, it was first introduced by Fritz von Wettstein in the 1920s as an experimental system in genetics research related to ploidy variation [13,14]. However, it was only after Engel’s mutagenesis studies in 1968 [15], that *P. patens* emerged as a widely used tool for genetic studies, primarily based on the Gransden strain (United Kingdom), of plant morphology, nutrition, the role and effect of different phytohormones in their responses to light and gravity, and other related studies [13,16]. Currently, beyond Gransden, other accessions/ecotypes have been isolated, such as Villersexel (France) and Reute (Germany), which show distinct genetic variation and phenotypes (e.g., different rates of sporophyte production).

This species gained even more prominence as a model organism due to its high homologous recombination, allowing precise gene targeting [17]. This achievement represented a breakthrough for plant research, enabling targeted genome manipulations comparable to those performed in classical model organisms such as yeast and mice [18]. The predominance of the haploid gametophyte in its life cycle constitutes another key advantage [19]. Since there is no masking of mutations in a heterozygous condition, mutant phenotypes can be observed directly, accelerating functional gene analyses. Furthermore, *P. patens* can be easily cultured in vitro, with rapid growth in simple media, high regenerative capacity from isolated cells, and maintenance under aseptic conditions, features that favor reproducible and low-cost experimentation [20].

This combination provides a valuable resource for investigating which genetic elements were inherited from common ancestors shared with green algae and which arose as innovations during terrestrial evolution. Subsequent studies, including chromosome-scale assembly, deepened the understanding of gene duplications, transposable elements, and overall genome organization, expanding opportunities for comparative analyses [11,21].

In biotechnology, *P. patens* stand out as a platform for the production of biopharmaceutical molecules, offering several advantages that include standard cultivation in bioreactors with low cost of maintenance when compared to other existing systems [22]. In addition, the high rate of homologous recombination allows multiple gene integrations or knockouts. In this context, *P. patens* comprises a cellular machinery for post-translational modifications that can be genetically engineered to synthesize human recombinant glycoproteins with optimized glycosylation patterns for therapeutic use [23]. Among the features already mentioned, this application has attracted increasing interest from the biopharmaceutical industry, particularly as a sustainable alternative to conventional mammalian cell systems [24]. Recently, two promising candidate moss-based biopharmaceuticals have advanced into the clinical development stage https://elevabiologics.com (accessed on 27 March 2026). Still, *P. patens* has also been amenable for metabolic engineering for the biosynthesis of valuable metabolites of commercial interest like very-long-chain polyunsaturated fatty acids and terpenoids [25]. Altogether, these important features make this species a strong candidate for contemporary synthetic biology purposes. *P. patens*’ robust molecular chassis is well suited for precise integration of gene networks/metabolic pathways and its use to evaluate other important topics in synthetic biology, like functional exploitation of synthetic promoters, is feasible [26,27].

From an ecological and physiological perspective, *P. patens* in an excellent experimental system for studies on environmental stress tolerance. Genes associated with the regulation of water balance, UV radiation, salinity, and low-temperature responses have been identified and characterized, highlighting the adaptive plasticity of this group [28]. This aspect is particularly relevant for understanding the molecular mechanisms that enabled the initial colonization of terrestrial environments by plants, as well as those underlying contemporary plant adaptation to climate change [29].

Thus, *P. patens* integrate three complementary dimensions: (i) a versatile functional genetic model comparable to other reference organisms; (ii) an evolutionary resource that sheds light on the transition of plants from aquatic to terrestrial life; and (iii) a biotechnological platform with direct applications in medicine, agriculture, and industry. The breadth of these uses consolidates its position as one of the most promising model systems in plant science in the twenty-first century [30,31].

### 2.2. The Physcomitrium patens Genome and Its Impact on the Scientific Community

The approximately 480 megabase pairs (Mbp) of the *P. patens* genome are distributed across 26 chromosomes and were the first moss genome to be decoded in 2008 (Gransden strain), establishing an unprecedented comparative basis with angiosperms and other groups of land plants [31,32]. The study revealed that about two-thirds of *P. patens* genes have orthologs in vascular plants, while also highlighting bryophyte-specific gene families that reflect both shared ancestry with green algae and innovations linked to terrestrial adaptation [33].

One of the most significant findings was the identification of genes related to abiotic stress responses, including those associated with desiccation, low temperature, and UV radiation [34]. These genes provide insights into the molecular mechanisms that enabled the transition of plants from aquatic to terrestrial environments, one of the major evolutionary transitions in the history of life [8].

Subsequently, the chromosome-scale assembly published by Lang et al. [9], provided even deeper insights into the genomic structure of the moss. This study identified large-scale gene duplication events, expansions of regulatory gene families, and the chromosomal distribution of transposable elements [35]. The availability of this refined resource enabled detailed comparative analyses with other plant lineages, paving the way for high-resolution phylogenomic investigations [8].

The recent near-telomere-to-telomere (T2T) genome assembly of *P. patens* (V6 genome, see https://phytozome-next.jgi.doe.gov/info/Ppatens_v6_1 (accessed on 1 April 2026) [36]), represents a major advance in bryophyte genomics by resolving previously inaccessible regions such as telomeres, centromeres, and highly repetitive sequences. This improved assembly redefines the chromosomal architecture of the species, confirming the presence of 26 chromosomes and uncovering a complex history of chromosomal breakage and fusion events [9]. In this context, previous studies suggest that the ancestral moss may have had seven chromosomes while the modern extant *P. patens* harbors a 26-chromosome configuration, generated by the two WGD rounds and events of chromosomal loss, break and fusion. Interestingly, whole genome duplication (WGD), an important evolutionary force that has shaped plant evolution, was suggested for other moss species, but evaluable genomic data from other bryophyte lineages indicate that the now sequenced *M. polymorpha* (liverwort) and *Anthoceros* (hornwort) lack evidence of WGD [9,37,38]. Indeed, the transition from a draft genome to a chromosome-level assembly was key to resolving scaffold fragmentation and achieving ordered chromosomes, which made it possible to reconstruct conserved collinear blocks, infer two rounds of WGD, and revise chromosome history with much greater confidence [36].

The *P. patens* genome served as an evolutionary reference for comparative studies, bridging the gap between charophyte algae and angiosperm genomes. Second, it consolidated the species as a functional genetic platform where hypotheses derived from genomic analyses could be experimentally tested through gene targeting. Finally, it stimulated new sequencing initiatives for bryophytes in general, expanding the representation of this group in genomic databases and strengthening our understanding of plant evolutionary diversity [8,17,19].

Thus, the *P. patens* genome not only expanded knowledge of moss evolution and biology but also redefined the role of this plant group within comparative genomics and biotechnology. Since its publication, the scientific community has gained access to a model organism capable of integrating evolutionary, functional, and applied perspectives, fundamentally transforming how mosses are studied [10,16].

### 2.3. Metabolic Genes in Physcomitrium patens

The genomic analysis of *P. patens* revealed a remarkably diverse set of genes related to primary and secondary metabolism, contributing to a broader understanding of metabolic evolution in land plants [10]. Among the most relevant aspects are genes associated with cell wall biosynthesis, secondary metabolite production, and hormonal regulation [39].

Regarding cell walls, *P. patens* exhibits a repertoire of genes encoding cellulases, hemicellulases, and expansins similar to those found in angiosperms, suggesting that the structural complexity of the plant cell wall was established early in plant evolutionary history [40]. Moreover, genes related to the biosynthesis of cutin and suberin molecules essential for desiccation protection were identified, indicating that these metabolic pathways played a key role in terrestrial colonization [41,42]. Another notable feature is the presence of complete biosynthetic pathways for classical phytohormones such as auxins, cytokinins, and abscisic acid (ABA). Although regulatory mechanisms may differ from those of vascular plants, the identification of these genes in *P. patens* confirms that hormonal signaling has played a central role since the earliest stages of plant terrestrial adaptation [43,44].

In terms of secondary metabolism, *P. patens* possess genes for the synthesis of flavonoids and phenolic compounds derived from the phenylpropanoid pathway—key metabolites in UV protection and defense against microorganisms [45]. Interestingly, although mosses do not produce true lignin, they possess enzymes involved in the polymerization of phenolic precursors, providing clues as to the evolutionary origin of this pathway characteristic of vascular plants [46].

The biotechnological potential of these genes has attracted growing interest. Functional studies have demonstrated, for example, that *P. patens* mutants deficient in flavonoid pathway genes exhibit increased sensitivity to oxidative stress, reinforcing the importance of these metabolites as natural antioxidants [47,48]. Additionally, the species’ capacity for precise genetic manipulation through gene targeting makes it an ideal platform for investigating specific gene functions within metabolic pathways, establishing direct connections between genomics and physiology [49]. Thus, the metabolic gene repertoire of *P. patens* not only clarifies fundamental aspects of land plant evolution but also offers concrete opportunities for biotechnological exploration in areas such as stress tolerance, metabolite production, and the synthesis of bioactive compounds [35].

### 2.4. Other Moss Genomes: Current Landscape and Future Perspectives

Although *P. patens* was the first and for a long time the only moss species with a fully sequenced and well-annotated genome, recent advances in next-generation sequencing technologies have greatly expanded access to the genetic resources of other mosses and other bryophyte lineages [50,51]. These efforts have been driven not only by the interest in understanding the evolutionary diversity of land plants but also by the search for novel genes and metabolic pathways with biotechnological potential [52].

In recent years, species such as *Sphagnum fallax* (H.Klinggr.) H.Klinggr. and *Sphagnum magellanicum* Brid. have had their genomes sequenced, revealing distinctive features related to carbon fixation and highlighting the crucial ecological role of peat mosses in regulating the global carbon cycle [53]. Similarly, the sequencing of the representative liverwort *Marchantia polymorpha* L., and hornworts from the genus Anthoceros (namely *Anthoceros agrestis* Paton and *Anthoceros punctatus* L.), provided new opportunities for comparative analyses among different bryophyte lineages [37,38,54,55].

Beyond these species, the genomes of *Ceratodon purpureus* (Hedw.) Brid. and *Pohlia nutans* (Hedw.) Lindb. have further enriched the bryophyte genomic landscape. The genome of *C. purpureus*, a cosmopolitan moss known for its exceptional tolerance to UV radiation, desiccation, and heavy metals, has revealed extensive expansions in gene families associated with DNA repair, oxidative stress responses, and hormonal signaling pathways [56,57]. These genomic traits underscore its ecological plasticity and have positioned *C. purpureus* as a promising model for environmental biotechnology, particularly in the development of bioindicators and phytoremediation strategies [58,59].

Meanwhile, *P. nutans*, a species widely distributed in polar regions and known for its resilience to freezing, desiccation, and high radiation levels, has emerged as an important extremophile model. Genome sequencing efforts have identified key genetic components involved in cryoprotection, antioxidant metabolism, and membrane stabilization under cold stress [57]. Transcriptomic and metabolomic analyses have further highlighted the production of bioactive molecules and antioxidant compounds, reinforcing the potential of *P. nutans* in applications in biotechnology and studies of stress tolerance mechanisms [60].

This growing collection of moss genomes underscores the importance of extending studies beyond *P. patens*, enabling comparative analyses that address both conserved traits and lineage-specific innovations [61]. The integration of these data sets into public repositories such as Phytozome and the NCBI Genome database has provided the scientific community with a solid foundation for exploring plant genome evolution on a broader scale [11]. To date, more than two hundred moss assemblies have been deposited in NCBI Genome https://www.ncbi.nlm.nih.gov/datasets/genome/?taxon=3208 (accessed on 1 April 2026). However, it is important to mention that this repository includes data that is not properly annotated, raw assemblies and other limitations that may lead to inconsistencies in the actual number of available genomes. In addition, a comprehensive super-pangenome initiative, the BryoGenomes database https://bryogenomes.org/ (accessed on 1 April 2026), hosts genomic data from 123 bryophyte species, including 82 mosses from multiple families, and offers substantial resources for exploring alternative evolutionary strategies for terrestrial colonization of plants and other molecular apparatus related to mosses and other bryophyte lineages. Thus, while *P. patens* established mosses as a model group for plant molecular research, the expanding genomic landscape now paves the way for a more comprehensive understanding of moss diversity and evolutionary plasticity. This broader context will be detailed in the next section, with emphasis on currently available genomes, the most recent developments, and future perspectives for moss genomics research [62,63].

## 3. Moss Genomes: Current Scenario

Over the past two decades, mosses have emerged as a key framework for understanding the origin of embryophytes, the evolution of ancestral metabolic pathways, and their close relationship with adaptation to extreme environments and the colonization of land [64]. The pivotal milestone was the sequencing of *P. patens*, which launched the bryophyte genomics era and triggered a cascade of discoveries [8,33]. Despite being a robust genomic reference, *P. patens* remained for a long time the only published moss genome, and even then, it was limited by short-read sequencing and fragmented assemblies that failed to resolve repetitive regions, centromeres, and large-scale structural rearrangements [65]. With the advent and consolidation of long-read platforms (PacBio/ONT), in combination with chromatin proximity data (Hi-C) and, in some cases, genetic maps, the field has been transformed, allowing chromosome-scale assemblies with unprecedented resolution for analyses of synteny, recombination, and 3D genome architecture.

**Figure 1:**
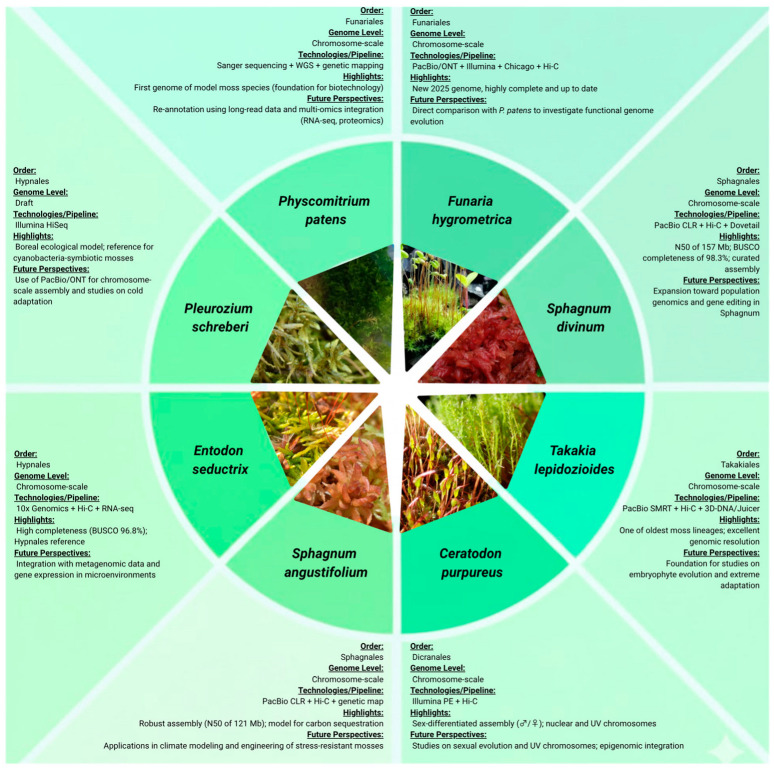
The diagram indicates representative genome assemblies from eight species from five different orders, summarizing their current genome assembly status and major biological insights. Each segment presents information related to particular genome assembly level (ranging from draft to chromosome-scale), sequencing technologies and scaffolding pipelines, based on data retrieved from the genome assembly publications cited in this review (e.g., Sanger, Illumina, PacBio CLR/SMRT, ONT, Hi-C, Chicago, genetic mapping, RNA-seq). Moreover, key genomic features from each species, as well as future research directions are also highlighted. Altogether, this profile illustrates the ongoing progress of genomic research within Bryophyta and its importance for plant evolution, functional genomics, ecological modeling and biotechnological applications. All moss photos were obtained from iNaturalist https://www.inaturalist.org/ (accessed on 8 April 2026), under CC0 license.

Recent large-scale pangenomic studies have demonstrated that mosses possess an unexpectedly expansive gene family space compared with vascular plants. Analyses incorporating dozens to hundreds of moss genomes revealed a high proportion of accessory and lineage-specific genes, reflecting distinct evolutionary strategies and remarkable functional diversity [66,67]. This extensive gene repertoire is likely linked to the long evolutionary history of mosses and their ability to adapt to diverse and often extreme terrestrial environments [40]. The comparative framework presented in Figure 1 illustrates the evolution of bryophyte genomics, emphasizing differences in genome assembly quality, sequencing technologies, and research perspectives among eight moss species.

This technological shift enabled testing of a long-hypothesized concept: mosses display a genomic continuum that juxtaposes high structural conservation—marked by strong synteny and collinearity—with pronounced functional plasticity [68]. Functional plasticity, particularly in gene-dense regions intertwined with transposable element (TE)-rich genomic compartments, has proven essential for enabling adaptation to harsh environments. Within this spectrum, Hypnales species (*Entodon seductrix* (Hedw.) Müll. Hal., *Hypnum curvifolium* Hedw.) represent the pole of chromosomal stability, characterized by ~1:1 collinearity and low evidence of recent gene duplication events. Conversely, *Sphagnum angustifolium* (Russow) C.E.O.Jensen, *Sphagum divinum* Flatberg & K.Hassel, and the desert mosses *Syntrichia ruralis* (Hedw.) F.Weber & D.Mohr and *Syntrichia caninervis* Mitt. represent the pole of adaptive plasticity, distinguished by high recombination rates, retrotransposon-enriched centromeres, and extensive genomic reorganizations [63,69,70,71]. This dichotomy reflects distinct evolutionary trajectories—acidic, oligotrophic peatlands versus deserts and arid habitats—but also suggests that the evolutionary gradient in mosses is directly shaped by environmental pressures that restructure chromosomes and interact with TE dynamics to consolidate genome-wide stability [72].

The availability of newly generated reference genomes, such as the chromosome-scale assembly of *Physcomitrellopsis africana* Broth. & Wager ex Dixon, has substantially expanded the taxonomic breadth of bryophyte genomic resources [73]. In parallel, comprehensive phylogenomic time-calibrated analyses have clarified deep evolutionary relationships among mosses, liverworts, and hornworts, resolving diversification patterns over approximately 500 million years [74]. These studies also revealed widespread gene tree incongruence, underscoring the complexity of early land plant evolution and the importance of dense taxon sampling in phylogenomic inference [75].

The strong collinearity observed between *Entodon* and *Hypnum* and the structural conservation found in *Funaria hygrometrica* Hedw. point to macroevolutionary constraints within basal embryophyte lineages [32,70]. However, such patterns must be interpreted cautiously in light of methodological biases: HiFi and Hi-C assemblies yield long contigs and resolve repetitive regions, whereas Illumina-based drafts tend to overestimate rearrangements. Consequently, the apparent “stability” of certain lineages may partially reflect technological advancements rather than purely evolutionary stasis. In contrast, Sphagnum species display widespread synteny breakdown and elevated recombination, associated with centromeres dominated by RLC5-Copia retrotransposons. Taken together, these findings indicate that genomic plasticity is an intrinsic property of peatland ecology—an environment where selective pressure, TE dynamics, and nuclear organization converge to shape structural genome evolution.

From a functional perspective, distinct and highly refined adaptive modules have emerged and some paramount questions could be addressed by the available moss genomic resources. As mentioned before, *P. nutans* is a remarkable species that thrives in extreme environments like Antarctica [60]. This moss exhibits expansion of genes involved in photoprotection and DNA repair, reinforcing the concept of convergent adaptive modules that integrate gene family expansion with regulatory flexibility. Moreover, the assemblies and annotations of *S. ruralis* and *S. caninervis* reveal targeted expansions of gene families involved in stress responses that collectively contribute to maintaining osmotic homeostasis, protein protection, and redox balance [69,76]. In this context, *S. caninervis*, a desert moss, and *S. ruralis*, a more widespread species, have complex molecular responses to desiccation that are shared and distinct between the two species. The expansion of key DT-associated gene families encoding protectants such as ELIPs has been documented in both species, of which a considerable portion appear to result from tandem duplications [77]. Transcriptomic analyses in both *Syntrichia* spp. have shown complex expression patterns with significant accumulation of ELIPs in response to desiccated conditions [69,77]. Similarly, late embryogenesis abundant (LEA) proteins, which are widely recognized for their role in plant desiccation tolerance, are highly accumulated in the desiccated tissues of *Syntrichia*. Yet, the adaptive gains reported are not limited to gene copy number increases: strong regulatory reprogramming has been detected, with promoters enriched in stress-responsive cis-motifs and transcription factors (e.g., MYB–ABI3) that modulate abiotic signaling cascades [76]. Therefore, the notable resilience to drought makes *Syntrichia* a valuable model to study the molecular apparatus involved in DT response in mosses [69,77].

The genome of the highly endangered *Takakia lepidozioides* S.Hatt. & Inoue, that belongs to the moss lineage estimated to have diverged about 390 mya, suggests that this species contains a particularly high number of fast-evolving genes under positive selection, including many stress-related genes to withstand severe environmental conditions [78,79]. *Takakia* dwells at high altitudes on the harsh Tibetan Plateau, experiencing severe freezing temperatures and extreme levels of UV-B radiation [78]. Notably, this species encompasses more phenylalanine ammonia-lyase (PAL) genes (20) than the model moss *P. patens* (16). PALs are key enzymes involved in the biosynthesis of phenylpropanoids and are highly expressed under UV-B stress treatments in *Takakia*, resulting in the production of a plethora of flavonoids and polyphenols—three to five times more than in *P. patens* [78]. Still, the gene expansion of nuclear-encoded pentatricopeptide repeat (PPR) proteins and RNA-editing sites are suggested to be related to UV-B radiation response, as expression of PPRs has been shown to be induced by UV-B treatments [78]. Finally, *Takakia* also features a specific genetic toolkit that enables efficient repair of DNA damage caused by intense radiation in its high-altitude habitat [78]. Collectively, these discoveries indicates that moss adaptation to adverse conditions extends well beyond the conservation of ancestral metabolic routes—it encompasses a comprehensive framework of biochemical innovations and functional modularity.

Another landmark is related to the cosmopolitan dioicous *C. purpureus*, a well-known model system for studying physiological responses to environmental stresses and, more recently, in evolutionary biology [56]. Genomic analyses revealed a massive UV sex chromosome system, significantly larger than its autosomes, comprising approximately 30% of both female and male genomes [56]. Different from other non-recombining systems, suppressed recombination in *Ceratodon* sex chromosomes shows minor degeneration and does not result in gene loss [56]. Conversely, these chromosomes have unusually high gene density (the U chromosome contains 3450 genes, while V has 3411) and high expression levels that are related to several regulators of sexual development [56].

Technological advances have been instrumental in this conceptual redefinition. The combination of long-read sequencing and Hi-C data has enabled highly complete assemblies (BUSCO completeness often >96%), while systems biology and functional genomics approaches—including scRNA-seq and integrative multi-omics—have unveiled cellular heterogeneity and functional compartmentalization of metabolic processes. Moreover, the integration of densely marker-saturated recombination maps with QTL (Quantitative Trait Locus) analysis has emerged as a crucial tool for elucidating the evolutionary mechanisms that underpin bryophyte diversification [80].

Nonetheless, the consolidation of this field continues to face significant conceptual and technical challenges. Most sequenced moss species are derived from single-accession data, limiting the ability to capture intraspecific structural variation such as CNVs, SNP diversity, and fine-scale population-level genetic variation. In addition, the lack of standardized pipelines for TE annotation, assembly quality metrics (N50, BUSCO, LAI), and consistent ortholog databases (Embryophyta vs. Eukaryota) hampers reliable cross-study comparisons. Furthermore, epigenomic and 3D structural data (methylomes, histone modification profiles, and TADs) remain scarce, yet are essential for integrating gene expression, recombination, and chromosome architecture.

Therefore, the future of moss genomics depends on a robust, collaborative comparative framework anchored on three core pillars: (i) the construction of telomere-to-telomere (T2T) pangenomes across multiple orders; (ii) the generation of integrated epigenomic and 3D chromatin maps to elucidate the role of chromatin in genome stability; and (iii) the implementation of multi-species single-cell multi-omics coupled with advanced genome editing to establish causal relationships between genomic variation and adaptive phenotypes.

In summary, the path forward for moss genomics requires this integrated and comparative program. Supported by chromosome-scale assemblies, advanced genetic editing tools, and multi-omics integration, this vision repositions mosses as central model systems at the intersection of evolution, ecology, and biotechnology.

## 4. Genome-Sequencing of Mosses: Recent Advances and Exploratory Potential

### 4.1. Advances in Sequencing Technologies and Methodological Approaches

The advent of next-generation sequencing (NGS) technologies, and more recently long-read platforms such as Pacific Biosciences and Oxford Nanopore, has revolutionized plant genomics by enabling chromosome-scale assemblies and high-quality gene annotations, even in organisms with complex genomes [36]. Within this context, mosses—particularly *P. patens*—have emerged as key model systems in modern plant biology, representing a crucial evolutionary link between green algae and vascular plants [10].

Table 1 summarizes the main genomic and sequencing features of selected moss species, including genome size, GC content, gene number, sequencing technologies, assembly pipelines, and genome assembly level. Assembly quality metrics (N50 and BUSCO) highlight differences in contiguity and completeness among species, reflecting the impact of distinct sequencing strategies and methodological approaches on genome quality in Bryophyta and the biological significance of the mosses with published genomes.

**Figure 2:**
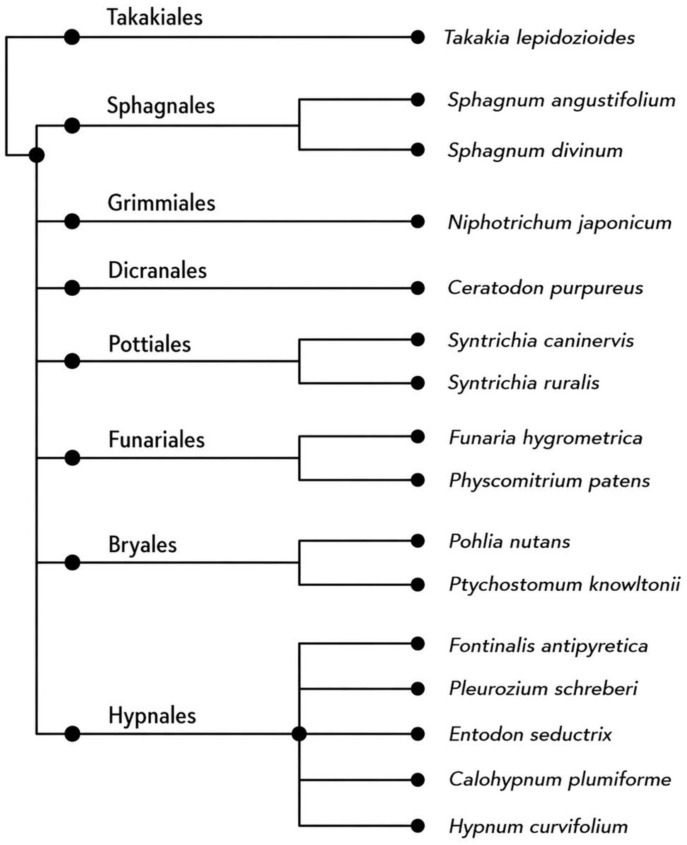
A cladogram to illustrate the phylogenetic relationship between the mosses with available genome assemblies. *Takakia lepidozioides* is the outgroup sister to all other mosses. It branches sequentially into Sphagnum (e.g., *S. angustifolium*, *S. divinum*), Bryopsida (including *Niphhotrichum japonicum* and *Ceratodon purpureus*), *Syntrichia* spp., Funariales (e.g., *Funaria hygrometrica*, and the model moss *Physcomitrium patens*), Bryales/Bryaceae (e.g., *Bryum argenteum*, *Ptychostomum knowltonii*), and Hypnales representatives, largest order of moss species.

Furthermore, as shown in Figure 2, a cladogram was generated to illustrate the phylogenetic relationship between the mosses with available genome assemblies.

The chromosome-scale genome assembly of *P. patens* [9] provided a detailed view of genome architecture and gene duplication events that predated the diversification of land plants. These genomic resources have revealed conserved metabolic pathways related to hormone biosynthesis, photosynthesis, and stress regulation, as well as ancient regulatory elements inherited from common ancestors [9]. With advances in molecular biology tools, *P. patens* became the first plant system where precise gene editing was extensively tested and optimized [9]. The application of CRISPR-Cas9 [86,87,88] and, more recently, prime editing technologies [89,90] established this moss as an experimental platform for large-scale functional gene studies and predictable genome engineering strategies. This capability is supported by its naturally high rate of homologous recombination—a rare feature among plants—which facilitates targeted gene replacement and loss-of-function analyses [10].

Recent breakthroughs include the use of single-cell RNA sequencing (scRNA-seq), which enables unprecedented resolution in studying cell differentiation and shoot apical meristem development [19,49]. These studies highlight the transcriptional complexity of mosses and reveal conserved regulatory gene networks shared with angiosperms. Parallel large-scale transcriptomic [91] and proteomic [92] datasets have contributed to comprehensive molecular atlases supporting functional and evolutionary investigations.

The cell wall biology of bryophytes has also gained prominence, with unique structural components and enzymes characterized in *P. patens* [92]. These findings offer new perspectives on the evolution of plant cell walls and biotechnological applications in biomaterials, resilience, and stress tolerance [39]. In parallel, initiatives such as the Space Moss Project, conducted by the Japan Aerospace Exploration Agency, have demonstrated the potential of mosses as model organisms for experiments on growth and adaptation in microgravity environments, expanding their role in space biotechnology and astrobiology.

Recent thematic reviews have advanced our understanding of bryophyte cell wall composition and function, with particular emphasis on arabinogalactan proteins (AGPs) as key molecular components. Studies in mosses and other bryophyte model systems, including *P. patens* and hornworts, indicate that AGPs display both conserved and lineage-specific structural features, suggesting roles in growth, development, and environmental interactions [93]. These findings provide important evolutionary perspectives on plant cell wall complexity and offer insights into the molecular mechanisms underlying bryophyte–microorganism interactions [39].

### 4.2. Comparative Genomics Across Moss Lineages

From an evolutionary perspective, recent discoveries reveal that moss genomes uniquely combine structural stability with functional plasticity. For example, species of the order Hypnales, such as *E. seductrix* and *H. curvifolium*, exhibit high gene collinearity and reduced rates of chromosomal rearrangement, indicating strong genomic conservation [70]. Comparative analyses among *P. patens*, *E. seductrix*, *H. curvifolium*, and *F. hygrometrica* demonstrate strong syntenic conservation across genomic blocks. Gene collinearity in Hypnales highlights the maintenance of ancestral arrangements of regulatory genes related to morphogenesis, cell wall metabolism, and hormonal signaling [9,32,70]. Functional syntenies were also observed in genes associated with the biosynthesis of phenolic compounds, flavonoids, and diterpenes, suggesting the existence of a pre-existing genomic organization of complex metabolic pathways for defense and cellular communication, conserved prior to the diversification of vascular plants [94].

In addition to these evolutionary dynamics, genomes of extremophilic mosses such as *C. purpureus* and *P. nutans* provide important examples of how distinct environmental pressures shape genomic architecture and function [57,58]. In *C. purpureus*, comparative analyses reveal expansions in gene families associated with DNA repair, oxidative stress responses, and hormonal signaling, reflecting its remarkable tolerance to UV radiation, desiccation, and heavy metal contamination [58]. This functional plasticity contrasts with the strong collinearity observed in Hypnales, suggesting that intense environmental pressures have favored lineage-specific genomic reorganizations and the diversification of metabolic pathways linked to environmental resilience. Meanwhile, *P. nutans*, predominant in polar regions, exhibits specialized gene sets related to cryoprotection, antioxidant metabolism, and membrane stabilization—traits aligned with its ability to withstand low temperatures and high radiation [57]. The presence of expanded metabolic pathways and evolutionary signatures of positive selection reinforces the notion that these extremophilic mosses represent lineages in which adaptive plasticity translates into both structural and functional genomic modifications, complementing the broader spectrum of genomic diversity observed across major bryophyte lineages [66,72,95].

In contrast, representatives of *S. angustifolium* and *S. divinum* deviate from this pattern, exhibiting low collinearity with *P. patens* and extensive chromosomal rearrangements [63]. These modifications are associated with structural plasticity and a highly dynamic genome architecture, with elevated recombination rates and centromeres enriched in retrotransposons—reflecting evolutionary strategies for adaptation to acidic peatland environments, conferring both genomic and physiological flexibility under environmental constraints [63].

### 4.3. Genomic Bases of Physiological Adaptation and Stress Response

Current moss genome assemblies have revealed a diversity of genes encoding antimicrobial peptides (AMPs) and cysteine-rich proteins (CRPs), arising from post-divergence gene duplications and neofunctionalizations within mosses [96,97]. In *P. patens*, different classes of AMPs and CRPs are induced under biotic and abiotic stresses and act synergistically with enzymes of the phenylpropanoid pathway, suggesting a role in defense responses [97,98]. Hence, plant-exclusive non-specific lipid transfer proteins (nsLTPs), are suggested to play important roles with in protection against desiccation, UV radiation, and fungal infections [96,99]. These AMPs are expressed under low-temperature and high-salinity conditions, possess structural motifs stabilized by disulfide bridges, and function as molecular mechanisms of resistance to environmental stress [100]. Another major finding was the identification of a functional biosynthetic gene cluster (BGC) associated with the synthesis of momilactones A and B, diterpenes with antifungal and allelopathic activity. This cluster, described in *Calohypnum plumiforme* (Wilson) Jan Kučera & Ignatov, was previously thought to occur only in grasses [70].

Additionally, the metabolic pathways described in mosses synthesize molecules of major pharmacological, cosmetic, and agronomic interest. Pathways responsible for the production of flavonoids and phenols—mediated by the gene families PAL, CHS (chalcone synthase), CHI (chalcone isomerase), and F3H (flavanone 3-hydroxylase)—were identified in *P. patens* and *Sphagnum* spp., exhibiting antioxidant and photoprotective functions with potential pharmaceutical applications [9,61]. Triterpenes and sterols were observed in *E. seductrix* and *Fontinalis antipyretica* Hedw.; these molecules are associated with mechanical resistance and pathogen defense, with potential use in biopolymers and sustainable biofilms [70]. Sulfated polysaccharides and exopolysaccharides produced by *Sphagnum* spp. demonstrate high water-retention capacity and carbon sequestration potential, being investigated as sustainable alternatives for agriculture and climate change mitigation [63].

In recent years, moss genomics has evolved into an integrated multi-omics approach, combining genomics, transcriptomics, proteomics, and metabolomics at single-cell resolution. This integration has enabled the functional validation of predicted genes in reference genomes such as *P. patens* and *S. angustifolium*, particularly those involved in secondary metabolite biosynthesis and stress response [80,91]. Single-cell RNA sequencing (scRNA-seq) in *P. patens* revealed transcriptional heterogeneity within morphologically uniform tissues, allowing the identification of specialized cell types responsible for polysaccharide secretion and phenolic compound accumulation. This confirmed that many antimicrobial peptides and enzymes of the phenylpropanoid pathway are expressed locally, suggesting functional compartmentalization of defense [91].

### 4.4. Emerging Biotechnological and Environmental Applications

This multi-omics integration has also expanded the understanding of ecological adaptation, showing that mosses developed genetic strategies based on targeted gene family expansions related to tolerance to severe environmental stresses. As mentioned before, in *S. ruralis* and *S. caninervis*, duplications in LEA, HSP (Heat Shock Protein), and ROS-detoxifying enzyme genes confer extreme tolerance to desiccation and UV radiation [77]. On the other hand, in the genus *Sphagnum*, the high recombination rates and extensive chromosomal rearrangements, as observed by Healey et al. [63], indicate structural innovation mechanisms linked to adaptation to peatland environments characterized by acidity, nutrient scarcity, and high concentrations of heavy metals. This genomic plasticity represents a balance between the stability of conserved syntenic blocks and localized adaptive evolution—a phenomenon comparable to that observed in bacterial extremophiles and symbiotic fungi [10].

Beyond elucidating evolution and adaptation, moss genomes have paved the way for highly versatile genetic platforms with applications in biotechnology and molecular bioengineering. *P. patens* has been consolidated as a stable plant bioreactor, efficient in producing recombinant proteins, vaccines, and therapeutic enzymes, due to its high rate of homologous recombination and ability to grow axenically at scale [10,16]. Furthermore, the characterization of modular metabolic pathways in *F. hygrometrica* and *S. divinum* provides new opportunities for the sustainable production of bioactive metabolites—antifungal, antioxidant, and biodegradable biofilm compounds [63].

The consolidation of moss genome assemblies marks a new era in plant genomics, establishing bryophytes as model systems for understanding the origin and evolution of embryophytes and revealing the coexistence of ancestral structural stability and adaptive genomic plasticity. Methodological advances, from the combination of long and short reads to integration with Hi-C and genetic maps, have elevated assemblies to chromosomal scale and enhanced the resolution of analysis of synteny, duplication, and transposable element dynamics. These genomes, now with high completeness (BUSCO > 96%), reveal a balance between conserved structural stability and adaptive innovation, reflected in the functional diversity of genes related to stress tolerance, metabolite biosynthesis, and antimicrobial defense. Future perspectives point to the continued integration of multi-omics and single-cell approaches, enabling the dissection of regulatory networks, metabolic modularity, and spatial gene expression in response to environmental conditions. In parallel, the biotechnological application of these genomes emerges as a promising frontier: mosses such as *P. patens*, *Sphagnum* spp., and *Syntrichia* spp. are becoming sustainable platforms for bioengineering, production of therapeutic peptides, and low-impact biopolymers. Thus, moss genomics transcends its descriptive origins to become a strategic field, connecting evolution, ecology, and green biotechnology in addressing the biological and environmental challenges of the 21st century.

## 5. Conclusions and Perspectives

As mentioned in this review, the moss *P. patens* is an outstanding model system for plant biology and a landmark tool for plant biotechnology. This species offers an attractive and advantageous toolkit chassis for synthetic biology due to its ease of growth and cultivation in bioreactors with inorganic liquid media, relatively low cost of maintenance when compared to other systems and supports highly efficient genetic engineering approaches (e.g., CRISPR/Cas9-based genome editing), which enables precise single-gene modifications as well as the integration or knockout of multiple genes. The *P. patens* platform offers a plethora of opportunities for the rapidly advancing field of synthetic biology regarding precise and predictable integration of multigene assemblies, due to its highly efficient homologous recombination rates, unprecedented in plant systems. For instance, this system has been demonstrated to be suited to testing short synthetic promoters to optimize expression.

Moreover, the sequencing of *P. patens* inaugurated a new era in bryophyte genomics, establishing an essential reference for understanding the early evolution of terrestrial plants. Building on this milestone, it has become possible to compare genome architectures across diverse lineages, including species inhabiting polar regions—ecosystems where mosses play critical roles in carbon cycling, soil formation, and resilience under extreme environmental conditions.

The rapid evolution of sequencing technologies—from Sanger-based approaches to long-read platforms integrated with Hi-C—enabled chromosome-scale assemblies and a refined understanding of synteny, gene duplication, and three-dimensional genome organization. These advances consolidated the view that mosses combine structural conservation with substantial adaptive plasticity, a feature particularly evident in species from polar and other extreme environments.

The results reviewed here reinforce the importance of mosses as models for investigating stress responses, metabolic evolution, and molecular mechanisms underlying adaptation to cold, desiccation, and high radiation. In this context, the genomic insights summarized in this work have direct implications for climate change research, as mosses serve as sensitive bioindicators and foundational components of polar ecosystems undergoing rapid transformation.

Currently, powerful tools such as scRNA-seq can potentially characterize the transcriptomic variations and dynamic gene expression patterns within specific cell types. As commented before, scRNA-seq has been used to unveil the dynamism of gene expression involved in the morphological transition between protonema (2D) and 3D gametophore development. However, a significant limitation related to this approach is the loss of spatial context due to tissue complexity. The integration of scRNA-seq with complementary profiling techniques such as Spatial Transcriptomics (ST) enables access to the full spectrum of genes simultaneously expressed within the gametophore. This combined approach reveals regulatory dynamics and spatially resolved, cell-type-specific gene activity associated with the growth transition, leading to insights that cannot be achieved through single-cell analysis alone. To date, these complementary technologies have been applied to map a dynamic transcriptional atlas during the life cycle of *Arabidopsis*.

Looking ahead, the field is poised to advance toward pangenome frameworks that capture intraspecific genomic variation, as well as the integration of epigenetic maps and chromatin architecture data. These approaches will be essential for linking genomic adaptations to ecological function, especially in regions heavily affected by ongoing climatic shifts. In fact, as the cost of genome sequencing continues to decline, a broad approach through pangenomics of single species or higher hierarchy phylogenetic clades can enable a consistent comprehension of core genes present in all members and the diversity of gene families that are unique and lineage-specific that can enrich our understanding through comparative genomics.

Thus, bryophyte genomics remains a strategic and expanding field, capable of illuminating both the evolutionary history of land plants and the biological challenges imposed by contemporary environmental change.

## Figures and Tables

**Table 1:** Genomic and sequencing features of selected moss species: The table presents genomic and sequencing data for various moss species. It includes the scientific name of each species (Species), the taxonomic order to which it belongs (Order), the genome size in megabases (Genome size, Mbp) and the total number of identified genes (Genes, n). Sequencing method indicates the technology or technologies used for DNA sequencing (e.g., Oxford Nanopore, PacBio SMRT, Illumina, Hi-C, 10× Genomics, RNA-seq), while Level refers to the completeness or organization of the genome assembly (e.g., Draft, Chromosomal, Near-T2T). N50/BUSCO provide assembly quality metrics, where N50 reflects contiguity (the contig or scaffold length at which 50% of the total genome is contained in contigs/scaffolds of equal or greater length) and BUSCO reflects completeness (percentage of highly conserved single-copy genes present in the assembly). Biological significance relates relevant features of individual mentioned species. References lists the corresponding bibliographic sources, when available.

Species	Order	Size (Mbp)	Genes (n)	Sequencing Method	Level	N50	BUSCO	Biological Significance	References
*Physcomitrium patens*	Funariales	~480	32,926	Sanger/WGS (v1) and Genetic Map	Chromosomal	2.8 Mb	-	Model organism in basic biology, biotechnology and synthetic biology	[8,9]
*Funaria hygrometrica*	Funariales	280 (Zurich) 314 (Uconn)	36,804 (Zurich) 36,301 (Uconn)	PacBio/ONT + Illumina + Chicago + Hi-C	Chromosomal	10.6 (Zurich) 8.47 (Uconn)	86.6% (Zurich) 82.7% (Uconn) 98.4% (Zurich/Viridiplantae) 92.9% (Uconn/Embryophyta)	Tolerance to heavy metals and bioremediation	[32]
*Ceratodon purpureus*	Dicranales	358 (♂ R40) 349.5 (♀ GG1)	30,425 (♂ R40), 30,425 (♀ GG1)	Illumina PE150 + Hi-C	Chromosomal	1.4 Mbp	69% (Embryophyta) 96.7% (Eukaryote, ♂), 96.4% (♀)	Sex-specific genetic architecture and response to environmental stresses	[56]
*Pohlia nutans*	Bryales	698.2	40,905	Illumina HiSeq X10	Draft	1.09 Mbp	83.9% (Viridiplantae)	Cold adaptation	[57]
*Sphagnum angustifolium*	Sphagnales	395	25,100	PacBio CLR + Hi-C Dovetail + Genetic Map (2.990 Markers)	Chromosomal	17.4 Mb	98.3% (Viridiplantae)	Global carbon cycling	[63]
*Sphagnum divinum*	Sphagnales	439	25,227	PacBio CLR + Hi-C Dovetail	Chromosomal	17.5 Mb	98.3% (Viridiplantae)	Global carbon cycling	[63]
*Syntrichia ruralis*	Pottiales	381.24	27,065	Illumina 2 × 150 + Transcripts	Chromosomal	24.41 Mpb	95% (Viridiplantae)	Desiccation tolerance	[69]
*Entodon* *seductrix*	Hypnales	348.4	25,801	10× Genomics + Hi-C + RNA-seq	Chromosomal	30 Mbp	96.8% (Viridiplantae)	Widespread moss in North America	[70]
*Hypnum curvifolium*	Hypnales	262	29,077	10× Genomics + Hi-C + RNA-seq	Chromosomal	20.7 Mb	97.2% (Viridiplantae)	Abundant moss species	[70]
*Syntrichia caninervis*	Pottiales	323.44	18,093	Oxford Nanopore + Hi-C + Illumina	Chromosomal	24.41 Mbp	98.1% (Eukaryota)	Desiccation tolerance	[71,77]
*Takakia lepidozioides*	Takakiales	325	27,467	PacBio SMRT + Illumina + Hi-C	Chromosomal	83 Mpb	97.3% (Eukaryota)/94.1% (Viridiplantae)/80.8% (Embryophyte)	Adaptations to environmental stress (UV-B and freezing)	[78]
*Calohypnum plumiforme*	Hypnales	335	32,195	PacBio SMRT + Illumina	Draft	790.02 Kbp	93.9% (Viridiplantae)	Momilactone-producing moss	[81]
*Fontinalis antipyretica*	Hypnales	385.2	16,538	BGISEQ-500 PE 150	Draft	45.8 Kbp	87.2% (Viridiplantae)	Aquatic lifestyle	[82]
*Niphotrichum japonicum*	Grimmiales	191.61	26,898	ONT 79 Gb + Hi-C 117 Gb + Illumina 120 Gb	Chromosomal	6.6 Mb	97% (Viridiplantae)	Heat tolerance	[83]
*Pleurozium schreberi*	Hypnales	318.34	15,992	Illumina HiSeq X (PE 2 × 150 bp)	Draft	204 Kb	90.1% (Eukaryote)	Key ecological functions and bioindicator of acid conditions	[84]
*Ptychostromum knowltonii*	Bryales	408.8	28,014	Long Reads + Hi-C	Chromosomal	32.61 Mb	92.2% (Viridiplantae)	Adaptations to extreme environments	[85]

## Data Availability

No new data were created or analyzed in this study.
